# Bridging autophagy and endolysosomal dysfunction: Role of bridging integrator 1 in Alzheimer’s disease

**DOI:** 10.4103/NRR.NRR-D-25-00243

**Published:** 2025-08-13

**Authors:** Julia Duckhorn, Doo Kyung Kim, Yu-Wen Alvin Huang

**Affiliations:** Department of Molecular Biology, Cell Biology and Biochemistry, Center for Translational Neuroscience, Carney Institute for Brain Science and Brown Institute for Translational Science, Brown University, Providence, RI, USA

**Keywords:** Alzheimer’s disease, amyloid-beta, autophagy, bridging integrator 1, cellular clearance, endolysosomal network, genetic risk factors, membrane dynamics, neurodegeneration, neuroinflammation, synaptic transmission, tau

## Abstract

Alzheimer’s disease is a devastating neurodegenerative disorder affecting millions worldwide, with current treatments offering only limited benefits. Central to emerging research is the role of autophagy and endolysosomal pathways, which are essential for clearing misfolded proteins and damaged organelles. Bridging integrator 1 (BIN1), traditionally recognized for its role in membrane remodeling and endocytosis, has recently emerged as a top genetic risk factor for Alzheimer’s disease, linking cellular clearance mechanisms to the development of toxic amyloid-beta plaques and tau tangles. In this review, we provide an accessible overview of how disruptions in autophagy and endolysosomal trafficking contribute to the neurodegeneration process in Alzheimer’s disease, positioning BIN1 as a central mediator within this complex network. Recent advances have shown that alterations in BIN1 expression and isoform distribution are associated with increased tau pathology and changes in amyloid-beta processing. Moreover, BIN1 appears to also influence synaptic transmission, neuroinflammation, and overall cellular homeostasis. The integration of recent findings not only deepens our understanding of Alzheimer’s disease pathology but also opens new avenues for the development of targeted treatments. This timely perspective underscores the potential of modulating BIN1 activity to enhance cellular clearance mechanisms and offers hope for more effective interventions for Alzheimer’s disease.

## Introduction

Alzheimer’s disease (AD) is a progressive neurodegenerative disorder that currently is the leading cause of dementia globally (No authors listed, 2024). Despite decades of research, current treatment options have limited efficacy in slowing or halting disease progression, underscoring the need for continued investigation into AD pathogenesis (Yiannopoulou and Papageorgiou, 2020). The severe cognitive impairments in AD are accompanied by hallmark pathological features, including cortical atrophy, the accumulation and spread of extracellular amyloid-beta (Aβ) plaques and neurofibrillary tangles (NFTs), inflammation, and impaired cellular homeostasis (Scheltens et al., 2021).

Growing evidence indicates that dysregulation of the endolysosomal and autophagic pathways is a key feature of AD pathology. These pathways are essential for maintaining cellular homeostasis by trafficking and degrading harmful materials such as misfolded proteins, damaged organelles, and toxic aggregates (Birgisdottir and Johansen, 2020). Disruptions in these networks are observed in many AD cases and are detected in the early stages of the disease, while several AD-associated genes have been linked to endolysosomal and autophagic function, further highlighting a critical link between these pathways and AD pathogenesis.

Among the genetic risk factors for AD, bridging integrator 1 (*BIN1*) has emerged as significant in several genome-wide association studies (GWAS) (Seshadri et al., 2010; Lambert et al., 2013; Jansen et al., 2019; Kunkle et al., 2019; Bellenguez et al., 2022). Since its identification as an AD risk gene, BIN1 dysfunction has been observed in AD patient brains and transgenic AD models, particularly linked to tau pathology, with numerous studies revealing its involvement in key cellular processes related to disease progression. Recent studies suggest potential interactions between BIN1 and endolysosome-autophagy network-related genes and proteins, indicating that BIN1 may contribute to the pathogenic dysregulation in AD, offering an exciting direction for further research into the mechanisms underlying disease pathogenesis (Bhattacharyya et al., 2022; Lambert et al., 2022; Jin et al., 2024; Zhang et al., 2024).

In this review, we provide an overview of the interconnected endolysosomal and autophagic networks, the modulations affecting their function at various process stages, and the impact on AD pathogenesis. We highlight the AD risk gene *BIN1* and its interactions with the endolysosomal-autophagic pathways, highlighting the potential mechanisms through which BIN1-dependent dysfunction contributes to the development and progression of AD.

## Data Sources

We conducted a comprehensive literature search using PubMed and Google Scholar to identify articles published in English that explore the role of BIN1 in endolysosomal and autophagy dysfunction and its implications for AD. Keywords included “BIN1,” “bridging integrator 1,” “Amph2,” or alternative nomenclature used for BIN1 in earlier studies, combined with terms such as “autophagy,” “endocytosis,” “lysosomal degradation,” “endolysosomal,” “Alzheimer’s disease,” and “protein trafficking.” Preference was given to articles published within the past ten years (2014–2024), with key studies outside this timeframe included when essential for context.

## Overview of Alzheimer’s Disease

AD is a debilitating neurodegenerative disorder that currently affects 55 million people globally, including one in three individuals above 85 years old, and is projected to impact more than 80 million people by 2030 (GBD 2019 Dementia Forecasting Collaborators, 2022). AD is marked by a progressive decline across multiple cognitive domains, including memory, mood and behavior, executive function, and motor abilities. These impairments diminish patients’ quality of life and place a substantial emotional and financial burden on families and communities throughout the disease (No authors listed, 2024). Existing treatments provide only modest benefits in mitigating disease progression, highlighting the urgent demand for novel therapeutic strategies (Yiannopoulou and Papageorgiou, 2020).

AD can be classified into two primary types: familial or early-onset AD, accounting for approximately 5% of cases, and sporadic or late-onset AD (LOAD), which represents the remaining 95% (No authors listed, 2024). Established susceptibility genes for early-onset AD include amyloid precursor protein (*APP*) and the presenilin genes 1 and 2 (*PSEN1*, *PSEN2*), with cognitive symptoms arising in patients around 30–40 years old (Ayodele et al., 2021). By contrast, LOAD typically manifests after age 65 and is influenced by a complex interplay of genetic and environmental factors, with age being the most significant risk factor (Sengoku, 2020). Several large-scale GWAS have identified over 80 loci associated with LOAD risk, identifying *APOE* as the most significant genetic risk factor, followed by *BIN1* (Seshadri et al., 2010; Lambert et al., 2013; Jansen et al., 2019; Kunkle et al., 2019; Bellenguez et al., 2022). Additional risk factors for AD include family history, as well as lifestyle factors such as diabetes, smoking, obesity, and education (Zhang et al., 2021). Together, these factors underscore the multifaceted etiology of AD.

Pathologically, AD is characterized by the accumulation and spread of extracellular Aβ plaques and intracellular NFTs formed from hyperphosphorylated tau (Kent et al., 2020). In the amyloidogenic pathway, APP is sequentially cleaved by β- and γ-secretases to produce Aβ peptides, primarily Aβ_40_ and the more aggregation-prone Aβ_42_. These peptides aggregate into oligomers, which further assemble into fibrils and eventually form extracellular plaques (Hampel et al., 2021). Aβ deposition typically precedes the emergence of clinical symptoms and tau pathology, with Aβ aggregates first appearing diffusely in neocortical regions before spreading to broader isocortical areas and the midbrain, with increasingly dense accumulation characterizes later stages of progression (d’Errico and Meyer-Luehmann, 2020). Tau, a microtubule-associated protein essential for axonal transport and neuronal structure, becomes hyperphosphorylated in AD, disassociating from microtubules and forming NFTs within neurons (Rawat et al., 2022). Tau pathology follows a separate, more stereotypical pattern of lesion distribution, with a later-stage acceleration that correlates more strongly to cognitive symptoms and visible degeneration. Early tau pathology is localized around the transentorhinal region, spreading to the entorhinal cortex and hippocampus, and ultimately affecting all hippocampal and isocortical areas (d’Errico and Meyer-Luehmann, 2020). Recently, researchers have suggested that tau pathology may have a prion-like spread across connected brain regions, with tau aggregates serving as the “seeds” driving the propagation of toxic intracellular aggregates (Colin et al., 2020).

The progressive accumulation and spread of these toxic aggregates are linked to additional pathological features, including neuronal and synaptic loss, neurotransmitter dysregulation, neuroinflammation, oxidative stress, and impaired protein homeostasis (Onyango et al., 2021; Cozachenco et al., 2023; Griffiths and Grant, 2023).

## Endolysosomal and Autophagic Pathways

**Figure 1:**
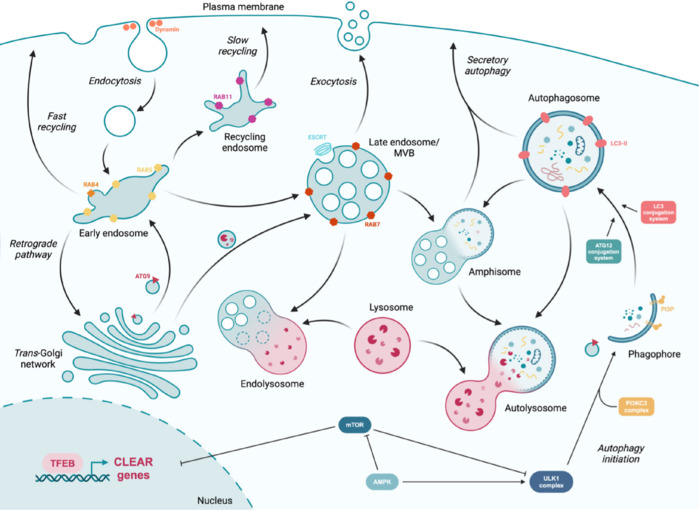
Schematic overview of the endolysosomal and autophagic pathways. In the endocytic pathway, extracellular materials are internalized and trafficked through early endosomes, which mature into late endosomes/MVBs. The autophagic pathway involves the formation of a phagophore that sequesters cytoplasmic components, developing into a double membrane-bound autophagosome. Subsequent fusion of autophagosomes, amphisomes, and late endosomes with lysosomes facilitates the degradation of enclosed substrates by lysosomal acid hydrolases. Created with BioRender.com. AMPK: Adenosine monophosphate-activated protein kinase; ATG: autophagy protein; LC3: light chain 3; mTOR: mammalian target of rapamycin; MVB: multivesicular body; TFEB: transcription factor EB; ULK1: Unc-51 like autophagy activating kinase 1.

The endolysosomal and autophagic pathways are highly conserved intracellular processes for the trafficking, sequestering, and degradation of unwanted cellular material (**Figure 1**). These pathways are critical for maintaining cellular health and protein homeostasis as they are responsible for the clearance of aggregated or misfolded proteins, damaged organelles, viral particles, and pathogens (Birgisdottir and Johansen, 2020). The function and coordination of endolysosomal and autophagic machinery is required for the clearance of both intracellular and extracellular material, which is notably defective in AD, characterized by the pathological aggregation and spread of Aβ plaques and NFTs.

### Endosomal system

The endosomal system consists of various vesicle compartments that regulate the trafficking and fate of intracellular and internalized materials. Endocytosis plays a crucial role in cellular functions, including the uptake and clearance of harmful extracellular material, regulation of membrane receptor signaling, and intracellular signal transduction (Sigismund et al., 2021). In neurons, endocytosis machinery is particularly important for signal transduction at dendritic and axonal branches distal from the cell body, as well as neurotransmitter reuptake (Roney et al., 2022). There are four primary types of endocytosis: clathrin-mediated endocytosis, caveolar endocytosis, macropinocytosis, and phagocytosis. Apart from the nonspecific macropinocytosis, these processes are initiated when cargoes, such as receptors, membranes, fluid, or viruses, bind to their corresponding cell surface receptors (Doherty and McMahon, 2009). The plasma membrane then forms invaginations through distinct, molecule-dependent mechanisms, leading to the internalization of the cargo.

Following invagination, the plasma membrane pinches off to form an intracellular vesicle, a process requiring the large GTPase dynamin (Prichard et al., 2021; Perrais, 2022). The internalized vesicle will subsequently fuse with an early endosome. The mildly acidic environment of the early endosome facilitates the dissociation of many cargoes from their receptors, allowing for their sorting and delivery to appropriate endocytic pathways (Jovic et al., 2010). Rab proteins, small GTPases (guanosine triphosphatases), play essential roles in vesicle trafficking and fusion during these processes. Rab5 regulates vesicle fusion and cargo sorting (Zhang et al., 2022). Cargo is recycled back to the plasma membrane via two primary pathways. In the fast-recycling pathway, regulated by Rab4, vesicles bud directly from the early endosome and fuse with the plasma membrane. In the slow recycling pathway, mediated by Rab11, vesicles are first transported to the perinuclear endocytic recycling compartment (ERC) before being routed to the plasma membrane (Cullen and Steinberg, 2018). Endosomal cargo destined for other cellular compartments is transported in a retrograde pathway to the trans-Golgi network (TGN) mediated by the retromer protein complex. Conversely, cargo marked for degradation is ubiquitinated and recognized by endosomal sorting complexes required for transport (ESCRT) family protein complexes (Norris and Grant, 2020).

This degradation pathway, largely controlled by Rab7, involves the budding of transport vesicles from the early endosome to fuse with late endosomes or the direct maturation of early endosomes into late endosomes (Huotari and Helenius, 2011; Borchers et al., 2021). Endosome maturation is characterized by both morphological changes and biochemical modifications, including progressive acidification driven primarily by the assembly and activation of V-ATPases. This acidification is vital for subsequent processes, including ligand-receptor dissociation, vesicle trafficking, and activation of hydrolytic enzymes in lysosomes (Chadwick et al., 2021). The maturation of early endosomes into late endosomes is also marked by the inward budding of the endosomal membrane, forming intraluminal vesicles (ILVs) (Huotari and Helenius, 2011). As maturation progresses, ILVs accumulate, becoming abundant in late endosomes and defining the structure of multivesicular bodies (MVBs). MVB formation occurs through two mechanisms: an ESCRT-dependent pathway, where Vps4 provides the energy to sever inward budding vesicles from the endosomal membrane, and an alternative pathway involving sphingomyelinases (Xu et al., 2023). MVBs have several fates, as they can fuse with lysosomes, where ILVs and their contents are degraded, or with autophagosomes, forming amphisomes that subsequently fuse with lysosomes. Additionally, MVBs can fuse with the plasma membrane, releasing ILVs into the extracellular space as exosomes (Gurung et al., 2021).

### Autophagy

Autophagy is a critical process for the sequestering, recycling, and degradation of unwanted cellular material at the lysosome. Autophagy has been implicated in numerous cellular processes, including development, inflammation, and cell cycle regulation, including axonal trafficking and synapse development in neurons (Klionsky et al., 2021). Most cells maintain a basal level of autophagy and upregulate autophagic processes as an adaptive response to various stress conditions. Autophagy can be further classified into macroautophagy (referred to as autophagy), chaperone-mediated autophagy (CMA), and microautophagy, based on distinct mechanisms of lysosomal delivery (Mizushima and Levine, 2020). The highly regulated autophagy process can be broken down into several conserved steps: autophagy initiation, phagophore nucleation, elongation, autophagosome maturation, autophagosome-lysosome fusion, and degradation.

Autophagy is triggered in response to many extracellular and intracellular stimuli, including nutrient starvation, oxidative stress, endoplasmic reticulum (ER) stress, infection, DNA damage, and organelle damage. Autophagy initiation is regulated by the phosphorylation of an Unc-51 like autophagy activating kinase 1 (ULK1) complex consisting of ULK1/2, ATG101, ATG13, and FIP200 (focal adhesion kinase family-interacting protein of 200 kDa) (Wei et al., 2024). The signaling kinases mammalian target of rapamycin complex 1 (mTORC1) and adenosine monophosphate-activated protein kinase (AMPK) regulate initiation through opposing phosphorylation of the ULK1 induction complex. In response to cellular stress, AMPK activation and/or mTORC1 inhibition triggers autophagy initiation through phosphorylation and activation of the ULK1 complex (Pareek and Kundu, 2024).

In addition to these cytosolic processes, autophagy is regulated at the transcriptional level. Transcription factor EB (TFEB) is a key transcription factor that regulates the induction and function of autophagy, along with other microphthalmia/transcription factor E (MiT/TFE) family members. TFEB is recognized as a master regulator of the coordinated lysosomal expression and regulation (CLEAR) network, a set of genes crucial for lysosome biogenesis and associated processes, including autophagy, lysosomal exocytosis, phagocytosis, and endocytosis (Tan et al., 2022). TFEB activity is tightly regulated through post-translational modifications such as phosphorylation and acetylation. Under nutrient-rich or resting conditions, TFEB is sequestered in the cytoplasm and remains inactive, notably through its phosphorylation by activated mTORC1 and interaction with 14-3-3 proteins (Takla et al., 2023). During nutrient deprivation or lysosomal stress, TFEB translocates to the nucleus, where it binds to CLEAR elements in target gene promoters, driving the synchronized activation of lysosomal and autophagy genes, enabling cells to adapt to stress and sustain efficient cellular clearance mechanisms (Tan et al., 2022).

Additional transcription factors, such as forkhead box protein O family proteins, E2F transcription factor 1 (E2F1), and tumor protein p53 have also been shown to regulate the expression of autophagy-related genes (Ma et al., 2022). Several epigenetic modifications have been shown to influence autophagy, such as histone methylation by co-activator-associated arginine-methyltransferase 1 (CARM1) and enhancer of zeste homolog 2 (EZH2), histone acetylation by Sirtuin 1 (SIRT1), and other modifications of histones and DNA (Shi et al., 2021; Shu et al., 2023). The coordination of the cytosolic signaling processes and transcriptional regulation is critical for balanced autophagic flux and proteostasis.

Activation of the ULK1 complex recruits a PI3KC3C1 complex (class III phosphatidylinositol 3-kinase complex I), made of vacuolar protein sorting-associated protein 34 (VPS34), VPS15, BECN1, ATG14, and NRBF2 (Majeed et al., 2022; Safaroghli-Azar et al., 2023). Activation of the PI3KC3C1 complex triggers phagophore nucleation and generates phosphatidylinositol 3-phosphate (PI3P). The PI3P generated on the phagophore facilitates membrane curvature and recruits effector proteins for further autophagic machinery recruitment (Palamiuc et al., 2020). Another PI3KC3 complex, PI3KC3C2 (class III phosphatidylinositol 3-kinase complex 2), has also been shown to potentially function in autophagy initiation as well as autophagosome maturation. The PI3KC3C2 complex contains the core VPS34, VPS15, and BECN1 subunits, but is UVRAG associated, and its activity is not autophagy specific (Safaroghli-Azar et al., 2023).

Autophagy regulation through the PI3KC3 complexes occurs through several interacting proteins, including inhibition through Bcl-2 and Rubicon (Run domain Beclin 1-interacting and cysteine-rich domain-containing protein) and activation through endophilin 1, autophagy and Beclin 1 regulator 1 (AMBRA1), and vacuole membrane protein 1 (VMP1) (Tran et al., 2021; Majeed et al., 2022; Safaroghli-Azar et al., 2023). Myotubularins such as myotubularin-related protein 14 and 3 (MTMR14 and MTMR3) can negatively regulate autophagy through dephosphorylation of PI3P to PIP, preventing autophagic progression (Palamiuc et al., 2020).

The autophagosomal membranes have been suggested to originate from various sources, including the ER, Golgi apparatus, mitochondria, endosomes, or newly synthesized membranes (Gomez-Sanchez et al., 2021). ATG9 vesicles originate at the ER and mature at the TGN under the regulation of the AP-4 complex, trafficking through the TGN and endocytic compartments, including early, recycling, and late endosomes (Holzer et al., 2024). Upon autophagy induction, they are directed to the nucleation site, where they serve as assembly platforms that recruit autophagy machinery and supply membranes and lipids essential for phagophore expansion.

The elongation of the phagophore is controlled by two ubiquitin-like conjugation systems. In the first, the E1 ubiquitin ligase ATG7 and E2 ubiquitin ligase ATG10 conjugate ATG12 to ATG5, which subsequently binds to ATG16L1, creating an ATG12–ATG5–ATG16L1 complex associated with the phagophore membrane. In the second ubiquitin-like system, LC3 (the microtubule-associated protein 1 light chain 3) is cleaved by ATG4 to form LC3-I. The E1-like enzyme ATG7 and E2-like ATG3, with the ATG12-ATG5-ATG16L1 complex, conjugate LC3-I to lipid phosphatidylethanolamine to form membrane-associated LC3-II (Mizushima, 2020).

While autophagy will typically involve random sequestering and uptake of cytoplasmic contents into phagophores, it often displays substrate specificity and will facilitate the degradation of specific cytosolic components. In selective autophagy, ubiquitinated targets are recognized by autophagy receptor proteins such as SQSTM1/p62, optineurin, NDP52, and NBR1 (Next to BRCA1 gene 1), which bind to ubiquitin-like modifiers in autophagosomes, such as ATG8 proteins LC3 and GABARAP, and ATG5 (Vargas et al., 2023). The ubiquitinated targets are then degraded in the autolysosome. Specific cellular targets are known to undergo selective autophagy in processes including mitophagy, ribophagy, ER-phagy (reticulophagy), lysophagy, and aggrephagy (Li et al., 2021).

The phagophore will expand and sequester cargo, eventually undergoing a membrane fission event to seal the cargo in a double membraned autophagosome. The mechanisms behind the membrane closure are unclear, with evidence suggesting the participation of ESCRT machinery and the ATG8/LC3 protein family (Jiang et al., 2021). Mature autophagosomes are transported along microtubules to the perinuclear region of the cell, where they will fuse with various endolysosomal compartments, including lysosomes, late endosomes, and MVBs to degrade their cargo (Lőrincz and Juhász, 2020; Zhao et al., 2021).

In addition to its degradative functions, autophagic machinery also facilitates a form of unconventional secretion, the secretion of cytosolic proteins lacking signal peptides that do not enter the conventional ER-Golgi pathway (Cavalli and Cenci, 2020). The secretory autophagy pathway has been linked to the release of proteins with extracellular roles in immune modulation, antimicrobial defense, tissue repair, and matrix remodeling. Additionally, impairments in autophagic or endolysosomal function increase the secretion of autophagy receptors and cargo associated with degradative autophagy, such as damaged organelles, pathogens, and protein aggregates, suggesting that autophagic vesicles and their cargo can be diverted away from the degradative pathway to protect the harmful accumulation of these proteins (Solvik et al., 2022). Where the pathways and machinery of degradative autophagy and secretory autophagy are shared and diverge is not well known. The secretory autophagosome is thought to be formed similarly to a degradative autophagosome, sharing processes of phagosome formation and autophagosome conjugation machinery (New and Thomas, 2019). Protein targets are engulfed by the expanding LC3 autophagosome or translocated into the intermembrane of a formed autophagosome. Specific SNARE proteins such as syntaxins 3/4 and SEC22B, Rab proteins such as Rab8, Rab11, Rab27, and Rab37, and other regulators are used to traffic the autophagosome to the plasma membrane and avoid lysosomal fusion (Leidal and Debnath, 2021; Lin et al., 2024). The autophagosome directly fuses with the plasma membrane for secretion, or fuses with a MVB before secretion, regulated by ESCRT machinery (Buratta et al., 2020).

The endosomal system and autophagy both rely on lysosomal activity for the degradation of cargo. Lysosomes serve as the final site of the cell for breaking down internalized materials, playing a crucial role in maintaining cellular homeostasis (Ballabio and Bonifacino, 2020). These organelles contain a wide array of acid hydrolases that become activated upon fusion with late endosomes, autophagosomes, and phagosomes, forming degradative compartments such as amphisomes, autolysosomes, endolysosomes, and phagolysosomes (Yim and Mizushima, 2020). The fusion of autophagosomes or amphisomes with late endosomes and lysosomes is a tightly coordinated process. It involves Rab GTPases like Rab7, SNARE proteins such as syntaxin 17, synaptosome-associated protein 29, and vesicle-associated membrane protein 8, and the homotypic fusion and vacuole protein sorting tethering complex (Lőrincz and Juhász, 2020). Beyond their intracellular roles, lysosomes are involved in processes such as membrane repair, degradation and remodeling of the extracellular matrix, and the release of non-degraded materials through lysosome exocytosis (Buratta et al., 2020; Tancini et al., 2020). To perform these diverse tasks, lysosomes must be highly dynamic in their localization. This mobility is facilitated by interactions with the extensive microtubule network, utilizing motor proteins such as kinesin and dynein for transport (Ballabio and Bonifacino, 2020).

Other forms of autophagy, CMA and microautophagy, share machinery with the endolysosomal and autophagy pathways. CMA involves the recognition and unfolding of target proteins containing a KFERQ consensus motif by cytosolic chaperones, notably HSPA8/HSC70. The target proteins are delivered across the lysosomal membrane through interaction with the CMA substrate receptor lysosome-associated membrane protein type 2A (LAMP2A) for degradation in the lumen (Yao and Shen, 2023).

In microautophagy, cytoplasmic substrates enter lysosomes or endosomes directly through engulfment or membrane deformation. Endosomal microautophagy occurs through the formation of MVBs and relies on ESCRT machinery and VPS4 to incorporate cargo into ILVs formed through membrane invagination. The MVB contents can then be degraded after lysosomal fusion (Yamamoto et al., 2023). This process can also be selective through the action of receptors such as HSPA8/HSC70 and NBR1 (Wang et al., 2023). Lysosomal microautophagy has also been shown to rely on ESCRT proteins and has been shown to be dependent on ATG proteins and Rab7 (Kuchitsu and Taguchi, 2024).

### Autophagy and endolysosomal dysregulation in Alzheimer’s disease

Dysfunction of the endocytic pathway is found among the earliest neuropathological hallmarks of AD. At preclinical or early stages of AD, neurons display enlarged Rab5-positive endosomes, along with elevated levels of endosomal hydrolases and key regulatory proteins such as Rab4, Rab5, Rab7, and MPR-46 (Cataldo et al., 1997, 2000; Mathews et al., 2002; Ginsberg et al., 2010a, b; 2011). These changes suggest an aberrant activation of the endocytic pathway, characterized by increased rates of cargo internalization and recycling.

Aggregated tau has been shown to enter cells via endocytosis, where it damages endolysosomal compartments and escapes into the cytosol, implicating endocytosis as a key mechanism in the pathological spread of NFTs (Frost et al., 2009; Wu et al., 2013; Polanco et al., 2018, 2021). The damaged Rab5-positive endosomes are recognized by galectin-8, initiating a pathway that directs cargo receptors to target aggregated tau for degradation via autophagy (Falcon et al., 2018). Additionally, exosomes have been identified as mediators of tau propagation and tau-containing exosomes have been found in the cerebrospinal fluid (CSF), plasma, and serum of AD patients (Zou et al., 2022). Exosomal tau released by neurons and microglia can initiate tau misfolding and aggregation in recipient cells, contributing to the propagation of tau pathology (Liang et al., 2023). Vesicle-associated tau was found to interact with the MVB marker TSG101, and Rab7 was shown to be involved in tau secretion, further implicating endolysosomal-associated compartments in the mechanisms of tau secretion (Yan and Zheng, 2021).

In the brain, β-secretase is predominantly localized to endosomes, and γ-secretase has also been found in endosomal compartments, suggesting a mechanism of increased amyloidogenic Aβ production in AD, especially as inhibition of endocytosis reduces Aβ generation, whereas enhanced endocytosis accelerates it (Koo and Squazzo, 1994; Huse et al., 2000; Mathews et al., 2002; Grbovic et al., 2003; Kinoshita et al., 2003; Carey et al., 2005; Wang et al., 2018). Improper sorting and excessive recycling of endosome-generated Aβ can also promote its exocytosis and extracellular accumulation (Andersen et al., 2005; Offe et al., 2006; Sullivan et al., 2011; Neuman et al., 2021). A small fraction of Aβ peptides is reported to be seen in MVBs and secreted in exosomes (Takahashi et al., 2002; Rajendran et al., 2006; Perez-Gonzalez et al., 2012).

An accumulation of autophagosomes was found in the dystrophic neurites of AD brains and in the APP/PS1 transgenic mouse model (Nixon et al., 2005; Yu et al., 2005). The neuronal autophagosome accumulation appears before visible neurodegeneration and Aβ deposition (Sanchez-Varo et al., 2012), suggesting the autophagic impairment is pathological and not a response to protein aggregation. Impaired autophagy has been further demonstrated by the increase of autophagy markers LC3B-II and SQSTM1/p62 in familial AD brains, colocalized with hyperphosphorylated tau, indicating impaired autophagosome clearance (Bordi et al., 2016). AD mice showed increased LC3 in astrocytes and increased p62 and ubiquitin in microglia (Pomilio et al., 2016).

Autophagy proteins ATG5, ATG16L, Rubicon, FIP200, BECN1, and NRBF2 levels have been shown to be reduced in AD brains (Pickford et al., 2008; Rohn et al., 2011; Lucin et al., 2013; Lachance et al., 2019; Heckmann et al., 2020). Conversely, autophagy is found to be transcriptionally upregulated in AD brains, potentially in response to autophagic impairment (Lipinski et al., 2010; Bordi et al., 2016). Disrupted lysosomal degradation has been indicated by changes in the level and cellular location of lysosomal proteases, hydrolases, and other lysosomal-related membrane proteins (Cataldo et al., 1991, 1995; Chai et al., 2019; Magini et al., 2015; Piras et al., 2016; Sjodin et al., 2019).

Hyperactivation of the autophagy inhibitor mTORC1 was found in AD brains, potentially contributing to impaired autophagy flux (Li et al., 2005; Sun et al., 2014). TFEB has also been shown to be dysregulated in AD, as reduced expression, nuclear localization, and transcriptional activity has been reported in AD patient brains, monocytes and lymphocytes, patient-derived cells, and cell models, correlating with disease severity (Tiribuzi et al., 2014; Reddy et al., 2016; Wang et al., 2016a; Heckmann et al., 2020). Other findings, however, have indicated increased TFEB levels or TFEB overactivation in patient-derived cells and mouse models (Zhang et al., 2012; Coffey et al., 2014; Landel et al., 2014; Bordi et al., 2016). Notably, glial cells in AD patients were shown to exhibit more pronounced TFEB nuclear staining compared to hippocampal neurons, and an upregulated lysosomal pathway is a hallmark feature of disease-associated microglial populations, illustrating the intricate, cell-specific dynamics of autophagic dysregulation in AD (Bordi et al., 2016).

Genetic autophagy inhibition in Atg7, Atg5, Becn1, or Nrbf2-deficient mice causes increased neurodegeneration associated with accumulating autophagy substrates and induced Aβ and tau accumulation (Hara et al., 2006; Pickford et al., 2008; Jaeger et al., 2010; Inoue et al., 2012; Nilsson et al., 2013; Cho et al., 2014; Lachance et al., 2019). CTSD depletion has been shown to lead to tau accumulation and neurodegeneration (Khurana et al., 2010). Autophagy activation through pharmacological inhibition of mTOR rescues Aβ and tau pathology in AD mouse models (Caccamo et al., 2010; Spilman et al., 2010; Majumder et al., 2011; Ozcelik et al., 2013). TFEB overexpression reduced pathological tau levels and rescues neurodegeneration in tauopathy mouse models (Polito et al., 2014; Xiao et al., 2014; Xiao et al., 2015b; Wang et al., 2016b). Overexpression of key autophagy proteins has been shown to rescue AD pathology, such as BECN1 (Pickford et al., 2008; Jaeger et al., 2010), NRBF2 (Yang et al., 2017; Cai et al., 2021), and the Rab7 activator CCZ1-MON1A (Cai et al., 2022).

APP has been shown to be a CMA substrate, and its interaction with HSPA8/HSC70 promotes APP degradation and decreases Aβ plaque accumulation in an APP/PS1 mouse model of AD (Park et al., 2016; Xu et al., 2021). Tau also interacts with HSPA8/HSC70 and has been shown to be degraded through CMA and endosomal microautophagy (Petrucelli et al., 2004; Wang et al., 2009; Caballero et al., 2018). Its targeting to different autophagy pathways has been shown to be regulated by blockages at the other pathways, potentially contributing to tau pathology in AD (Caballero et al., 2018).

The most frequent mutations associated with early-onset AD, *APP*, *PSEN1*, and *PSEN2*, have been linked to various endolysosomal and autophagic impairments in addition to their role in Aβ production (La Rosa et al., 2015; Cacace et al., 2016; Hung and Livesey, 2018; Kwart et al., 2019). PSEN1-deficient cells exhibit increased mTOR activation, reducing autophagy induction and limiting transcription of genes within the TFEB-regulated CLEAR network (Reddy et al., 2016). PSEN1 also regulates lysosome proteolysis and autophagosome-lysosome fusion (Wilson et al., 2004; Lee et al., 2010, 2015; Bustos et al., 2017; Hung and Livesey, 2018). *PSEN2* mutations have also been shown to impair autophagy, disrupt lysosome proteolysis, and reduce autophagosome-lysosome fusion (Neely et al., 2011; Fedeli et al., 2019).

While the etiology of LOAD is more complex, several large-scale genomic analyses have identified numerous genetic risk factors (Seshadri et al., 2010; Lambert et al., 2013; Jansen et al., 2019; Kunkle et al., 2019; Bellenguez et al., 2022). Many of these identified genes have been shown to participate in the regulation and function of the endolysosome and autophagy pathways, including *PICALM* (Ando et al., 2022), sortilin-receptor 1 (*SORL1*) (Barthelson et al., 2020), clusterin (*CLU*) (Satapathy and Wilson, 2021), CD2‐associated protein (*CD2AP*) (Tao et al., 2019), Ephrin A1 (*EPHA1*) (Tanabe et al., 2011), Src homology domain-containing inositol 5′-phosphatase 1 (SHIP1/*INPP5D*) (Yeoh and Krebs, 2023), histone acetyltransferase 8 (*KAT8*) (Yoo et al., 2024), phospholipase D3 (PLD3) (Paumier and Gowrishankar, 2024), ubiquitin-like protein ubiquilin-1 (*UBQLN1*) (Zheng et al., 2020), granulin (*GRN*) (Simon et al., 2023), and *CTSD* (Drobny et al., 2022).

## Bridging Integrator 1

BIN1 (also known as amphiphysin 2, amphiphysin II, Amph2, BRAMP2, ALP1, and SH3P9) is a member of the BAR (Bin/Amphiphysin/Rvs) domain protein superfamily, which is primarily involved in the formation and detection of local membrane curvatures (Ren et al., 2006). BIN1 plays critical roles in numerous cellular functions across various tissues, including endocytosis, membrane remodeling, cytoskeletal regulation, and cell cycle progression. Disruptions in BIN1 have significant medical implications, as it has been linked to cancer progression, multiple myopathies, and heart failure (Prokic et al., 2014).

**Figure 2:**
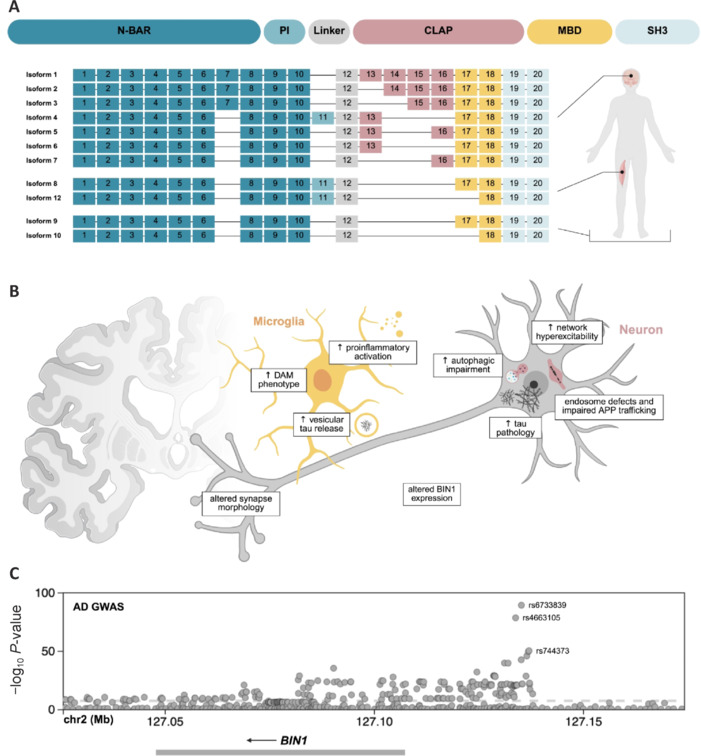
Genetic architecture and pathological relevance of *BIN1* in Alzheimer’s disease. (A) BIN1 contains several key protein structures, including an N-BAR domain, a PI binding motif, a CLAP binding domain, a MBD, and a SH3 domain. The *BIN1* gene is divided into 20 exons that undergo alternative splicing events that result in diverse tissue-specific isoforms. (B) Summary of the potential contributions of BIN1 to AD pathogenesis. Altered *BIN1* expression in AD is associated with tau pathology, endolysosomal and autophagic defects, disrupted APP trafficking, synaptic and network dysfunction, and glial activation with proinflammatory features or phenotypes of DAM. (C) Regional Manhattan plot showing AD GWAS association signals at the *BIN1* locus, highlighting the most significant SNPs. Data are extracted from the dataset of Bellenguez et al. (2022). Created with BioRender.com. AD: Alzheimer’s disease; APP: amyloid precursor protein; BIN1: bridging integrator 1; CLAP: clathrin and AP2; DAM: disease-associated microglia; GWAS: genome-wide association studies; MBD: Myc-binding domain; N-BAR: Bin/Amphiphysin/Rvs with an N-terminal amphipathic helix; PI: phosphoinositide; SH3: Src homology 3.

The *BIN1* gene contains 20 exons, which are spliced into at least 11 isoforms (**Figure 2A**). Isoforms 1 through 7 are brain-specific, isoform 8 is muscle-specific, and isoforms 9 and 10 are ubiquitously expressed (Butler et al., 1997; Leprince et al., 1997; Ramjaun et al., 1997; Tsutsui et al., 1997; Wechsler-Reya et al., 1997; Ren et al., 2006; Prokic et al., 2014). The human *BIN1* promoter region contains muscle regulatory sites, including myogenin/myoD, Sp1, and serum response factor (SRF), along with a conserved nuclear factor kappa B binding site in both human and mouse genomes (Mao et al., 1999). Additionally, p53 (Merlo et al., 2014), E2F1 and other E2F family transcription factors (Kinney et al., 2008; Cassimere et al., 2009; Kumari et al., 2015), c-MYC, and MYC-interacting zinc finger transcription factor 1 (MIZ1) (Pyndiah et al., 2011) have been identified as modulators of *BIN1* transcription. Several splicing factors have been identified to regulate *BIN1* splicing, including serine and arginine rich splicing factor 1 (SRSF1) (Anczukow et al., 2012), serine and arginine rich splicing factor 2 (SRSF2) (Sha et al., 2024), quinone oxidoreductase 1 (NQO1) (Du et al., 2023), muscleblind like splicing regulator 1 (MBNL1) (Fugier et al., 2011), and on-POU domain containing octamer binding (NONO) (Hu et al., 2020).

BIN1 isoform expression in the central nervous system is complex and dynamic across cell types and developmental stages. In the human brain, oligodendrocytes and microglia express the highest BIN1 levels, while isoforms 1 and 9 are the most abundant transcripts and are found in both neurons and glia (Crotti et al., 2019; Taga et al., 2020; Gabitto et al., 2024). BIN1 isoforms 1, 2, 3, 5, and 7 are expressed in both neurons and astrocytes, with neurons primarily expressing higher molecular weight isoforms, particularly neuron-specific isoform 1, while astrocytes predominantly express isoforms 9 and 10 (Zhou et al., 2014). Microglia mainly express isoforms 6, 9, 10, and 12, with isoform 10 being the most abundant, a pattern consistent across human and mouse microglia, including human induced pluripotent stem cells (iPSC)–derived microglia (Crotti et al., 2019; Taga et al., 2020; Sudwarts et al., 2022). BIN1 expression in oligodendrocytes, predominantly composed of lower molecular weight isoforms, such as isoform 9, which is found distributed across both grey and white matter, increases during development and maturation (De Rossi et al., 2016, 2019). BIN1 expression also changes dynamically during lineage specification: in neurons, higher molecular weight isoforms (1, 2, 3, 5, and 7) increase and peak at the final neuronal stage, while microglial isoforms (6, 9, 10, and 12) transiently rise at the NPC stage before declining (Lambert et al., 2022; Hu et al., 2025). In contrast, higher molecular weight isoforms decrease and microglial isoforms increase during microglial differentiation (Hu et al., 2025), highlighting BIN1 isoform regulation as a dynamic process shaped by development and cell fate decisions.

BIN1 contains several key protein structures, including an N-BAR domain, a phosphoinositide (PI) binding motif, a clathrin and AP2 (CLAP) binding domain, a Myc-binding domain, and a Src homology 3 (SH3) domain. The N-BAR domain, encoded by exons 1 to 10, is expressed in all isoforms and plays a critical role in sensing and generating membrane curvature (Peter et al., 2004). The PI binding motif, encoded by exon 11, is present only in a few BIN1 isoforms (isoforms 4, 8, and 12) and targets BIN1 to membrane compartments such as T-tubules, which are muscle-specific plasma membrane invaginations (Lee et al., 2002). The CLAP binding domain, encoded by exons 13–16, is found exclusively in brain isoforms and is responsible for binding to the endocytic proteins clathrin and AP2 (Ramjaun and McPherson, 1998). The Myc-binding domain, encoded by exons 17 and 18, and the SH3 domain, encoded by exons 19 and 20, are present in all isoforms, with the SH3 domain known to bind proline-rich motifs (Sakamuro et al., 1996; Owen et al., 1998; Ramjaun and McPherson, 1998).

Amphiphysins, including BIN1 and its mammalian homolog amphiphysin 1 (AMPH1), have been primarily associated with endocytosis across neuronal and non-neuronal cells, as well as endosome trafficking and recycling (see below). The ubiquitously expressed BAR domain is able to bind lipid membranes and induces membrane curvature (Peter et al., 2004), such as T-tubules in muscular cells (Lee et al., 2002; Fu and Hong, 2016), liposomes, and plasma membranes (Picas et al., 2014; Wu and Baumgart, 2014; Adam et al., 2015; Gowrisankaran et al., 2020). BIN1 has also been proposed to regulate the actin cytoskeleton and microtubule network, suggesting a coordinated role in remodeling both membrane and cytoskeleton structures (Meunier et al., 2009; Falcone et al., 2014; Hong et al., 2014; D’Alessandro et al., 2015; Drager et al., 2017; Picas et al., 2024).

Initially identified as a tumor suppressor, BIN1 promotes cell death in malignant cells (Chen et al., 2023). Nuclear BIN1 isoforms interact with the transcription factor c-MYC, known for its roles in cell growth, apoptosis, and malignancy, and have been shown to inhibit c-MYC-driven transformation (Sakamuro et al., 1996; Elliott et al., 1999, 2000). BIN1 is further implicated in oncogenic pathways and cell cycle regulation through its involvement in Rb-induced cell cycle arrest and interactions with the DNA-binding factor E2F1 (Kinney et al., 2008; Cassimere et al., 2009; Kumari et al., 2015; Folk et al., 2019), facilitation of Fas/FasL-mediated apoptosis (Esmailzadeh et al., 2015), mediation of growth arrest and senescence through regulation of the proto-oncogene BRAF activity (Wajapeyee et al., 2008), and interaction with the proto-oncogene c-ABL (Kadlec and Pendergast, 1997). Beyond cell cycle control, nuclear BIN1 participates in DNA repair and associated senescence pathways through interactions with PARP1 (Pyndiah et al., 2011), E2F1-dependent regulation of ATM and the MRN complex (Folk et al., 2019), and binding to the Ku70/Ku80 complex (Ramalingam et al., 2007) and XRCC4 (Grelle et al., 2006).

BIN1 additionally regulates calcium levels and localization of the L-type calcium channel Ca_v_1.2 in muscle and neurons (Hong et al., 2010; Hong et al., 2012; De La Mata et al., 2019). Recent studies have also linked BIN1 to synaptic transmission and neuronal electrical activity, highlighting its role in maintaining neuronal firing homeostasis, calcium regulation, and gene expression (De Rossi et al., 2020; Schurmann et al., 2020; Voskobiynyk et al., 2020; Saha et al., 2024). The role of BIN1 in modulating immune and inflammatory responses is found broadly (Muller et al., 2005; Chang et al., 2007; Wang et al., 2017; Thomas and Prendergast, 2023) and in the central nervous system (Sudwarts et al., 2022; Ponnusamy et al., 2023).

### BIN1 genetic and functional association with Alzheimer’s disease

The connection between BIN1 and AD is well-established, having been first identified in GWAS, where *BIN1* ranks second only to *APOE* in genome-wide significance (**Figure 2B**; Seshadri et al., 2010; Lambert et al., 2013; Almeida et al., 2018; Jansen et al., 2019; Kunkle et al., 2019; Bellenguez et al., 2022). *BIN1* was found to be a top hit in several epigenome-wide association studies examining DNA methylation patterns in AD patient brain tissues, including those from preclinical stages (De Jager et al., 2014; Yu et al., 2015), suggesting epigenetic changes occur early in the disease process. The AD-associated *BIN1* risk alleles are predominantly upstream and are non-coding, indicating they likely regulate *BIN1* expression (**Figure 2C**).

BIN1 expression has been shown to be altered in AD, with studies reporting changes at both the transcript and protein levels. BIN1 protein levels are elevated in plasma, while *BIN1* mRNA levels are increased in peripheral blood mononuclear cells of AD patients (Sun et al., 2013). Similarly, *BIN1* transcript levels were shown to be elevated in the frontal cortex of AD patients (Chapuis et al., 2013; Martiskainen et al., 2015). Another study reported that although no association was found between *BIN1* expression and AD pathology, elevated expression of *BIN1*, and specifically isoform 1 was associated with later age at disease onset and shorter disease duration (Karch et al., 2012). Although AD risk SNPs at the *BIN1* locus (rs744373 and rs59335482) were not initially linked to changes in *BIN1* expression (Karch et al., 2012), later studies associated these variants to increased *BIN1* mRNA levels in the brain (Chapuis et al., 2013; Martiskainen et al., 2015). Additionally, the *BIN1* risk SNP rs35103166 has been linked to increased expression of lower molecular weight isoforms (9, 10, and 12) in peripheral blood (Hu et al., 2025). The SNP rs744373 has also been linked to increased hippocampal *BIN1* expression and severe memory impairment in temporal lobe epilepsy (Bungenberg et al., 2016). In contrast, reduced BIN1 protein levels have been observed in AD patients (Glennon et al., 2013; McKenzie et al., 2017), while BIN1-immunoreactive neuropil areas are decreased in the AD brain, despite an increase in the number of neurons with detectable BIN1 expression (Adams et al., 2016).

Emerging evidence suggests that these expression changes are isoform-specific. In gray matter from AD brains, isoform 1 protein levels are decreased, alongside an upregulation of lower molecular weight isoforms (De Rossi et al., 2016), a pattern further supported by findings of reduced isoform 1 and increased isoform 9 expression in AD brain tissue (Holler et al., 2014). A later study also reported reduced isoform 1 protein levels in the frontal cortex and hippocampus of AD samples, along with differential downregulation of isoform 1 transcripts in the MSBB dataset (Marques-Coelho et al., 2021). Marked reductions in cytoplasmic BIN1 were observed in AD brain tissue (Glennon et al., 2020), consistent with the loss of neuronal BIN1 isoforms, which are predominantly localized to the cytoplasm. Similarly, a significant reduction in neuronal BIN1 isoform 1 expression was observed at the onset of disease symptoms in a mouse tauopathy model (McAvoy et al., 2019). Higher expression of *BIN1* isoforms 6, 9, or an intron-retaining transcript was associated with increased Aβ pathology, while higher isoform 1 expression was linked to reduced Aβ burden, consistent with the isoform-specific changes seen in AD (Yu et al., 2015), while conversely, another study found no change in the *BIN1* splicing profile in the temporal cortex relative to tangle pathology (Martiskainen et al., 2015). The pathological significance of isoform-specific BIN1 dysregulation remains unclear, particularly whether these alterations act as drivers or consequences of neurodegeneration, and how they may be influenced by cell type. Growing evidence suggests a critical role for microglial BIN1 in AD pathogenesis, as two AD risk variants at the *BIN1* locus, rs6733839 and rs13025717, are localized to microglia-specific enhancer regions and directly modulate BIN1 expression in microglia (Nott et al., 2019; Corces et al., 2020; Novikova et al., 2021; Young et al., 2021; Cooper et al., 2022).

BIN1 has been implicated in both Aβ and tau pathology in AD. Methylation at specific CpG sites within the *BIN1* locus has been associated with both Aβ load and tau tangle density (Yu et al., 2015). However, findings on the involvement of BIN1 in Aβ pathology have been less consistent than those related to tau. *BIN1* expression in brain samples was found to be positively associated with β-secretase activity (Martiskainen et al., 2015), while expression of *BIN1* isoform 1 was associated with higher levels of amyloid pathology (Karch et al., 2012), suggesting a possible link to amyloidogenic APP processing and Aβ pathology. BIN1 also has been shown to accumulate adjacent to amyloid deposits in various AD transgenic models and in human AD brain tissue and various AD transgenic models (De Rossi et al., 2019). BIN1 depletion increased Aβ production and secretion in mouse and human cells (Bali et al., 2012; Miyagawa et al., 2016), with the strongest effect observed in axons (Ubelmann et al., 2017), a phenotype that was rescued by the re-expression of neuronal BIN1 (Ubelmann et al., 2017). In iPSC-derived cerebral organoids, BIN1 depletion was reported to reduce APP β-CTF (Lambert et al., 2022), although another study in iPSC-derived models found no significant changes in APP, β-CTF, or Aβ levels (Saha et al., 2024). BIN1 overexpression decreased Aβ_40_ levels in SH-SY5Y cells, but its knockdown did not alter Aβ levels (Glennon et al., 2013). Similarly, BIN1 depletion in multiple mouse models did not affect Aβ levels (Andrew et al., 2019). In Drosophila, BIN1iso1-induced neurotoxicity was unaffected by APP expression (Lambert et al., 2022), and Aβ_42_-induced neurotoxicity was unaffected by expression of the BIN1 ortholog Amph (Chapuis et al., 2013), consistent with other findings suggesting a limited or inconsistent relationship between BIN1 and Aβ pathology.

BIN1 has been more consistently associated with tau pathology. Levels of BIN1, particularly isoform 9, were shown to correlate with NFT pathology, but not with diffuse or neuritic amyloid plaques in AD brains (Holler et al., 2014). The most reported *BIN1* AD risk variant, the SNP rs744373, was shown to be significantly associated with elevated tau-PET signal but not Aβ-PET (Franzmeier et al., 2019). Some studies did not find significant associations between *BIN1* SNPs, including rs744373 and rs59335482, and clinical, pathological, or biomarker measures of AD (Kauwe et al., 2011; Karch et al., 2012; Martiskainen et al., 2015), but later research has reported variants such as rs59335482 were associated with tau pathology but not Aβ load in AD patients (Chapuis et al., 2013), while other variants, including rs744373 and rs13031703, have been linked to elevated tau and phospho-tau levels in the CSF of individuals with mild cognitive impairment or AD, also without association with Aβ pathology (Wang et al., 2016c). Further studies report that *BIN1*-associated SNPs such as rs6431223 and rs6733839 correlate with increased phospho-tau levels in the CSF (Crotti et al., 2019) and higher Braak NFT stages (Shade et al., 2024), also without association with amyloid deposition or Aβ burden. BIN1 has been reported to colocalize with NFT pathology (Holler et al., 2014), although others found no significant concordance (Chapuis et al., 2013; Adams et al., 2016; De Rossi et al., 2017). BIN1 and tau have been detected colocalized in seeding-competent extracellular vesicles isolated from CSF of AD patients (Crotti et al., 2019).

Direct interactions between BIN1 and tau have been reported in multiple models (Chapuis et al., 2013; Sottejeau et al., 2015; Malki et al., 2017; Lasorsa et al., 2018; Sartori et al., 2019; Glennon et al., 2020), potentially influencing tau aggregation, propagation, and neurotoxicity. In Drosophila, the BIN1 paralog Amph was shown to regulate tau-induced neurotoxicity, but not Aβ42-induced toxicity (Chapuis et al., 2013; Dourlen et al., 2017). In tau-expressing human cells and tau transgenic mouse models, BIN1 expression was found to promote phospho-tau levels and exacerbate tau pathology (Thomas et al., 2019a), whereas Sartori et al. (2019) found BIN1 expression reduced intracellular tau inclusions and attenuated tau pathology. Extending these findings, Ponnusamy et al. (2023) examining the effects of BIN1 loss in specific cell types have revealed additional complexity. BIN1 depletion in forebrain neurons and oligodendrocytes reduced tau accumulation in the hippocampus, entorhinal/piriform cortex, and amygdala in tau transgenic mice, but exacerbated pathological tau burden in the somatosensory cortex and spinal cord, suggesting both cell type and region-specific effects of BIN1 on tau pathology. While BIN1 modulation in iPSC-derived cerebral organoids had no significant effect on tau, BIN1 depletion in human-induced neurons led to increased total and phospho-tau levels (Saha et al., 2024), suggesting a protective role for endogenous neuronal BIN1. This aligns with findings from rat neurons, where neuronal BIN1 reduced intracellular tau aggregation and limited tau propagation, while the ubiquitous isoform 9 had no effect (Calafate et al., 2016; Glennon et al., 2020). In contrast, BIN1 depletion in microglia reduced vesicular tau release and phospho-tau spread, while overexpression of isoform 9 exacerbated tau pathology (Crotti et al., 2019), highlighting the differential effects of BIN1 on tau pathology according to isoform and cellular context, with non-neuronal isoforms like isoform 9 potentially promoting pathology, unlike the protective influence seen with neuronal BIN1. Additional findings have shown that BIN1 and tau co-localize with the actin cytoskeleton (Sottejeau et al., 2015; Drager et al., 2017), suggesting that BIN1-tau complexes disrupt the interaction between actin and microtubules. They also co-localize with CLU, another significant AD risk gene, at the cytoskeleton, with increased BIN1-CLU interaction observed in AD brains (Zhou et al., 2014), further supporting potential mechanistic links between BIN1 and tau pathology.

BIN1 regulates the expression and localization of Ca_v_1.2, the dominant L-type voltage-gated calcium channel in the hippocampus, resulting in disrupted calcium dynamics and neural network dysfunction in human neurons, correlated with reduced synapse numbers and the dysregulated expression of synaptic transmission and ion transport genes (Saha et al., 2024). In rodent models, reduced tau levels were found to weaken the interaction between BIN1 and Ca_v_1.2 (Voskobiynyk et al., 2020), highlighting the potential role of BIN1 in calcium regulation as a contributing factor to AD pathogenesis.

DNA repair and cell cycle pathways are mechanistically linked, as DNA damage can trigger aberrant cell cycle re-entry in postmitotic neurons, leading to apoptosis or other forms of neurodegeneration. Markers of DNA damage, such as γH2AX, are elevated in AD, alongside dysregulation of DNA repair pathways involving several BIN1-linked proteins, including ATM, the MRN complex, Ku, DNA-PKcs, PARP1, and XRCC4 (Wong and Chow, 2023). In parallel, aberrant activation of cell cycle and fate regulators such as the Rb/E2F pathway, also associated with BIN1, has been observed in AD (Mao and Zhang, 2022), suggesting BIN1 may contribute to AD pathogenesis through these pathways.

Growing evidence supports a fundamental role for neuroinflammation in the progression of AD pathology, with BIN1 as a potential regulator in this context. BIN1 depletion increased inflammation during aging in mice (Chang et al., 2007). BIN1 modulates the expression of indoleamine 2,3-dioxygenase 1 (IDO1), a key enzyme in activating tryptophan metabolism (Muller et al., 2005). Elevated levels of IDO1 have been identified in microglia, astrocytes, and neurons of AD hippocampal sections and co-localize with plaques and tangles (Sharma et al., 2022). BIN1 has been shown to regulate proinflammatory activation and cytokine production in microglia, as well as AD-associated transcriptomic changes (Sudwarts et al., 2022; Ponnusamy et al., 2023). These results show the multifaceted role of BIN1 in regulating microglial activation during neuroinflammation and AD pathology.

## Bridging Integrator 1 in Endolysosomal Pathways

### Clathrin-coated vesicle formation and trafficking

The role of BIN1 in clathrin-mediated endocytosis (CME) has been established through its interactions with endocytic proteins, subcellular localization, and functional studies. BIN1, such as Amphiphysin I, is enriched at the nerve terminal (Ramjaun et al., 1997; Wigge et al., 1997). BIN1 interacts with dynamin, synaptojanin, and clathrin, core components required for membrane remodeling, vesicle scission, and clathrin coat formation during CME (Ramjaun et al., 1997; Ramjaun et al., 1999; Dong et al., 2000; Nemoto et al., 2001). BIN1 has also been shown to interact with other CME-related proteins, including endophilin and the components of the AP2 adaptor complex (Leprince et al., 1997; McMahon et al., 1997; Micheva, Kay, et al., 1997; Micheva et al., 1997b; Ramjaun and McPherson, 1998). Additionally, BIN1 can form heterodimers with amphiphysin I, another BAR domain protein, and this complex has been shown to regulate CME via dynamin recruitment (Wigge et al., 1997). In COS cells, coexpressing BIN1 and amphiphysin I increases endocytosis, measured by transferrin uptake (Wigge et al., 1997), while BIN1/amphiphysin I double knockout in cultured mouse neurons exhibited significant deficits in synaptic vesicle endocytosis and recycling (Di Paolo et al., 2002). Through its BAR domain, BIN1 likely contributes to membrane curvature and tubulation, as well as sense membrane curvature at clathrin-coated pits, and recruits dynamin and synaptojanin to facilitate vesicle scission (Casal et al., 2006; Meinecke et al., 2013; Wu and Baumgart, 2014; Adam et al., 2015; Fujise et al., 2021). BIN1 also interacts with actin and the actin nucleation promoter WASP, both essential for membrane invagination (Hong et al., 2014).

In rat hippocampal neurons, BIN1 depletion increased endocytic flux and transferrin uptake, with a corresponding enlargement of Rab5-positive early endosomes and an increase in activated, membrane-associated Rab5 (Calafate et al., 2016). Overexpression of isoform 1 reduces transferrin uptake and the number of Rab5-positive endosomes, consistent with suppressed endocytosis and endosomal trafficking (Calafate et al., 2016; Zhang et al., 2024). BIN1 interacts with Rab5 through the guanine nucleotide exchange factor (GEF) RIN3, an activator of Rab5 (Shen et al., 2022). RIN3 was shown to interact with BIN1 through its SH3 domain and recruited BIN1 into Rab5-positive endocytic vesicles (Kajiho et al., 2003; Shen et al., 2020). The RIN3/Rab5-positive vesicles did not contain the early endosome marker EEA1, suggesting BIN1 interacts with endolysosomal machinery early in the endocytic pathway, potentially regulating transport from the plasma membrane to the early endosome. In Neuro2A cells, BIN1 isoform 1 and isoform 9 were shown to bind RIN3 at similar levels, consistent with their shared SH3 domain (Bhattacharyya et al., 2022). Despite this, RIN3 was shown to recruit neuronal isoform 1, but not isoform 9, to Rab5-positive early endosomes, indicating isoform-specific and cell-type specific effects on the endocytic pathway.

The BIN1/RIN3 interaction is significant in the context of AD, as it influences APP processing and Aβ generation by modulating the endocytic trafficking of APP and the β-secretase BACE1. APP is internalized via clathrin-mediated endocytosis, while BACE1 follows an Arf6-mediated pathway into early endosomes (Limone et al., 2022). Normally, APP is targeted for lysosomal degradation, while BACE1 recycles to the plasma membrane, maintaining minimal Aβ production. RIN3-dependent recruitment of BIN1 isoform 1 inhibits APP endocytosis, but not BACE1 endocytosis, spatially separating these proteins and reducing Aβ generation (Bhattacharyya et al., 2022). A GWAS study has linked AD risk to the SLC24A4/RIN3 locus, and elevated RIN3 expression was observed in APP/PS1 transgenic mice. RIN3 was also shown to promote phosphorylation of tau and βCTF production, further implicating the BIN1/RIN3 interaction in AD pathogenesis (Shen et al., 2020).

### Non-clathrin-coated endocytosis and phagocytosis

Fast endophilin-mediated endocytosis (FEME) is a clathrin-independent endocytic pathway critical for the rapid retrieval of synaptic vesicles, membrane proteins, and the endocytosis of various receptors, including G-protein-coupled receptors (GPCRs) and receptor tyrosine kinases (Casamento and Boucrot, 2020). Unlike classical CME, FEME relies on actin dynamics and the BAR domain properties of endophilin to drive membrane invagination and fission, bypassing the need for a clathrin coat. BIN1 is recruited to FEME priming patches at the plasma membrane through interactions with CIP4 and lamellipodin (Lpd) and is localized on most FEME priming patches and carriers (Ferreira et al., 2021). The role of BIN1 in FEME appears to involve generating membrane curvature and facilitating scission. Additionally, BIN1 recruits Dynein to FEME carriers under the regulatory influence of Cdk5 and GSK3β, supporting the scission and retrograde trafficking of FEME carriers, which depend on the microtubule motor Dynein (Ferreira et al., 2021).

Research also indicates that BIN1 is involved in the regulation of phagocytosis, which is vital for immune response and homeostasis. BIN1 has been shown to bind to α integrins, which are essential for the immune response and phagocytic activity (Wixler et al., 1999). Studies have also demonstrated that BIN1 is crucial for the phagocytic function of macrophages, where it facilitates the uptake of apoptotic cells and pathogens (Gold et al., 2000, 2004). Upon engagement of a phagocytic receptor, rapid actin polymerization occurs beneath the particle, initiating the engulfment process. This step is followed by the activation of PI-3 kinase, which recruits BIN1 to the phagosome. BIN1 is essential for the subsequent recruitment of dynamin, facilitating membrane extension around the particle and its internalization (Gold et al., 2000). Under normal circumstances, BIN1 transiently associates with phagosomes and dissociates early in their maturation. However, in the case of *C. pneumoniae*, BIN1 remains indefinitely on the vacuole. This aberrant retention on the immature phagosome prevents its progression to a fully functional phagolysosome, allowing the bacterium to evade macrophage-mediated killing and persist within host cells (Gold et al., 2004), suggesting BIN1 is critical for both membrane elongation and the coordination of phagosome maturation.

### Endosome trafficking

BIN1 has also been implicated in endolysosomal regulation through its role in protein trafficking from early endosomes. In HeLa cells, transfected BIN1 was shown to colocalize on intracellular vesicles with various endolysosomal proteins, including the early endosome markers EEA1 and SNX4, as well as the late endosome marker CD63, the lysosomal marker LAMP1, and the ER marker calnexin (Leprince et al., 2003).

Expression of human BIN1 in *Drosophila* photoreceptor neurons induced isoform-specific endosomal defects and neurodegeneration (Lambert et al., 2022). Isoform 1 expression led to the abnormal accumulation of single-membrane vesicles in the cytoplasm preceding neuronal death. These BIN1-induced vesicles were positive for endolysosomal markers, including Rab5, PI3P-binding FYVE domains, Rab7, and the exosome/MVB marker Evi (Evenness Interrupted), implicating dysfunction in the endolysosomal pathway. The BIN1-induced neurodegeneration was rescued by Rab5 and Rab11 expression, as well as by one of the Rab4 constructs tested, indicating that disruptions occurred at the level of early endosome trafficking. In human iNs, isoform 1 overexpression also led to enlarged early endosomes and degeneration, while isoform 9 expression reduced endosome volume (Lambert et al., 2022). Additionally, BIN1 depletion in iNs reduced the size of EEA1-expressing early endosomes, an effect reversed by isoform 1 expression, highlighting the role of BIN1 isoform 1 in regulating early endosome processes. The ability of Rab11, and potentially Rab4, to rescue endosomal dysfunction in *Drosophila* photoreceptor neurons further suggests that BIN1 impacts endosomal recycling.

BIN1 has been shown to regulate the endolysosomal trafficking of BACE1 after endocytosis. BIN1, through its ubiquitous BAR domain, directly binds BACE1 (Miyagawa et al., 2016). BIN1 depletion in HeLa cells caused enlarged EE1A-positive early endosomes and was shown to reduce BACE1 trafficking from the EE1A-positive early endosomes to lysosomal compartments, impairing degradation (Miyagawa et al., 2016). In neuronal cultures, depletion of BIN1 resulted in deficient BACE1 recycling, specifically in axons (Ubelmann et al., 2017; Perdigao et al., 2021). BIN1 was shown to colocalize with BACE1 at Rab5-positive early endosomes, showing preferential overlap in axonal endosomes to dendritic endosomes (Ubelmann et al., 2017). BIN1 depletion caused the accumulation of BACE1-containing tubular carriers from early endosomes (Ubelmann et al., 2017), suggesting a critical role for BIN1 in facilitating tubule scission necessary for BACE1 recycling to the plasma membrane.

It has been suggested that BIN1 might bind to early endosome membranes to sense or induce curvature for scission through its recruitment of dynamin or EHD1 (Ubelmann et al., 2017). EHD family proteins play key roles in membrane tubulation and vesicle fission, and coordinating the endocytic trafficking of early and recycling endosomes (Naslavsky and Caplan, 2011). Notably, the loss of EHD1 function impairs the recycling and axonal sorting of internalized BACE1, leading to reduced Aβ production (Buggia-Prevot et al., 2013). BIN1 has been shown to interact with EHD1 and regulate endocytic trafficking, potentially through the recruitment of EHD1 to endosomal membranes (Pant et al., 2009; Posey et al., 2014). This interaction may position BIN1 as a key regulator of EHD1-mediated processes, further linking BIN1 to the modulation of BACE1 recycling and AD pathology.

In both neuronal and non-neuronal cell models, BIN1 depletion was shown to increase BACE1 protein levels and promoted Aβ secretion (Miyagawa et al., 2016), suggesting that BIN1’s involvement in endolysosomal trafficking is not limited to the neuronal isoform 1 and is distinct from its role in endocytosis. Conversely, another study reported that isoform 1, but not isoform 9, suppressed intracellular Aβ generation (Ubelmann et al., 2017). These differences may stem from the increased complexity of endocytic trafficking in polarized cells, where specialized endosomes are required to ensure the precise sorting of cargo to distinct cellular compartments. Notably, many endosomal markers, such as Rab8 and EEA1, are absent from axonal endosomes, and distinct sets of motor proteins regulate endosomal trafficking to axons or dendrites (Schmidt and Haucke, 2007). In polarized cells, particularly neurons, BIN1’s role in endosomal trafficking may be more specialized compared to its function in non-polarized cells through interactions with axon-specific endosomal components and motor proteins These findings underscore the need for further research to elucidate the isoform-specific effects of BIN1 on endolysosomal trafficking and Aβ pathology.

### Exocytosis

EHBP1L1, another EHD protein, interacts with BIN1 at the endocytic recycling compartment (ERC) to regulate recycling exocytosis (Nakajo et al., 2016). A Rab8-binding protein complex consisting of EHBP1L1, BIN1, and dynamin localizes to the ERC and facilitates the generation of vesicles and tubules containing apical cargo proteins. In small intestine organoids, the depletion of EHBP1L1 and BIN1, or the inhibition of dynamin, impairs protein sorting. Additionally, the downregulation of Rab10, along with BIN1 and dynamin, impairs erythroblast enucleation (Wu et al., 2023). In EHBP1L1-deficient mice, mislocalized nuclei and mitochondria were observed in muscle fibers, a pathology resembling centronuclear myopathy caused by mutations in BIN1 or dynamin 2, further implicating the functioning of these proteins within the same cellular pathways.

In rodent primary neuron cultures, postsynaptic BIN1 strongly colocalizes with Rab11 and Arf6, small GTPases known for regulating membrane protein trafficking through recycling endosomes (Schurmann et al., 2020). Rab11 also participates in exocytosis and neurite outgrowth, involving interactions with other Rab proteins, such as Rab8 and Rab10 (Homma and Fukuda, 2016). Arf6 also regulates recycling endocytic traffic, as well as exocytosis, actin cytoskeleton and plasma membrane dynamics, and phosphoinositide signaling (D’Souza-Schorey and Chavrier, 2006). Knockdown of BIN1 disrupts the size and organization of Rab11/Arf6 structures in postsynaptic compartments, leading to a buildup of vesicles in dendritic spines (Schurmann et al., 2020). This likely reflects impaired recycling from endosomes to the plasma membrane, suggesting that BIN1 participates in the Rab11/Arf6 recycling exocytosis process. The loss of BIN1 expression in neurons decreased vesicular release probability and caused an accumulation of docked vesicles, along with an increased number of reserve pools of synaptic vesicles, suggesting disrupted synaptic vesicle turnover. In hippocampal synapses of *Bin1* cKO mice, the density and organization of presynaptic machinery were altered, including key proteins such as Bassoon, Synaptoporin, and Munc13-1, which regulate presynaptic vesicle dynamics (De Rossi et al., 2020). These findings collectively point to the critical role of BIN1 in regulating exocytosis mechanisms essential for proper synaptic transmission.

The role of BIN1 in recycling exocytosis is also found in *Caenorhabditis elegans* (*C. elegans*), where mutation of the BIN1 ortholog AMPH-1 caused defective recycling of membrane proteins (Pant et al., 2009). AMPH-1 also interacts with RME-1 (the *C. elegans* ortholog of EHD1), which is known to regulate Arf6-dependent exocytosis (Caplan et al., 2002), as well as BIN1 activity during T-tubule formation in myoblasts (Posey et al., 2014). Certain ERC proteins, including EHBP1L1, partially localize to late endosomes and lysosomes, highlighting a spatial and functional connection between the ERC and lysosomes. Under lysosomal overload stress, Rab8 and Rab10 are stabilized on lysosomes, where they cooperate with EHBP1 and EHBP1L1 to regulate stress-induced lysosomal enlargement and secretion (Eguchi et al., 2018). EHBP1 promotes endosomal tubulation through interactions with the actin cytoskeleton (Wang et al., 2016d), while EHBP1L1, in collaboration with BIN1, generates membrane curvature necessary for vesicle excision at the ERC (Nakajo et al., 2016). In polarized epithelial cells deficient in Rab8, EHBP1L1, BIN1, or dynamin, apical cargo proteins accumulate in lysosomes (Nakajo et al., 2016). This evidence suggests that BIN1, together with EHBP1L1 and dynamin, could play a critical role in vesicle formation and budding on lysosomal membranes, maintaining lysosomal morphology and function under stress conditions and contributing to lysosomal exocytosis.

Further insights come from studies on *Drosophila*. At the larval neuromuscular junction (NMJ), the BIN1 ortholog Amph is enriched at postsynaptic regions and facilitates the exocytosis of the cell adhesion molecule Fasciclin II (FasII) (Mathew et al., 2003). In *Amph* mutants, FasII integration into the synaptic membrane is reduced, although internalization remains intact, suggesting impaired exocytosis. Additionally, the knockdown of *Amph* inhibits pore stabilization during exocytosis, resulting in irreversible pore expansion and collapse-like exocytosis (Biton et al., 2023). These findings underscore the conserved role of BIN1 in vesicle dynamics and membrane cycling across species.

Exosomes have recently been implicated in tau propagation, with tau-containing exosomes isolated from AD patient CSF, cultured neurons, and cellular tau models. These exosomes transfer tau between neurons through synaptic junctions, inducing tau aggregation in recipient cells (Jackson et al., 2022). BIN1 has been shown to play a critical role in ESCRT-dependent microvesicle formation and release, initially identified in cardiomyocytes (Xu et al., 2017). The ESCRT-III protein CHMP4B is recruited to BIN1-enriched membranes, where the interaction between BIN1’s ubiquitous BAR domain and CHMP4B facilitates vesicle formation and release into the extracellular space. Beyond cardiomyocytes, BIN1-mediated EV release also occurs in neurons and microglia within the central nervous system. In the context of AD, BIN1-associated EVs from microglia contain tau protein and exhibit seeding capacity, contributing to tau propagation (Crotti et al., 2019). Tau-containing EVs from AD patient CSF samples also include BIN1 and have been shown to induce tau seeding *in vitro*. Overexpression of isoform 9 in HEK293T cells increases EV-associated tau release and exacerbates tau pathology, while neuronal isoform 1 did not. Notably microglial-specific knockout of BIN1 significantly reduces tau levels in EVs and diminishes tau spreading in PS19 mice, implicating microglial BIN1 in the packaging of tau into EVs (Crotti et al., 2019).

## Bridging Integrator 1 in Autophagic Pathways

### Autophagy initiation

In a recent study, BIN1 knockdown in mouse hippocampal neurons led to elevated autophagic flux, indicated by elevated levels of LC3-II and BECN1, alongside reduced SQSTM1/p62 (Jin et al., 2024). The BIN1 knockdown also promoted the nuclear translocation of TFEB. BIN1 depletion reduced AKT activity, inhibiting its ability to sequester TFEB in the cytoplasm through phosphorylation, allowing TFEB to translocate into the nucleus. Once in the nucleus, TFEB increases the expression of numerous autophagy and lysosomal genes, including unc-51 like kinase 3 (*ULK3*), an autophagy initiator regulating autophagosome formation, linking BIN1 knockdown to increased autophagy independently of MTOR.

### Phagophore nucleation and elongation

BIN1 engages the autophagy-initiating PI3KC3 complex: a BIN1 monoclonal antibody (99D) co-immunoprecipitates with BECN1 and colocalizes with BECN1 and SQSTM1/p62 in human cell lines (Thomas et al., 2019a, b). Beyond this cytosolic role, the TGN has emerged as a contributor to autophagosome membrane formation (De Tito et al., 2020). The clathrin adaptor protein complex AP-1 is essential for the budding of LC3-associated membrane domains from the TGN, which contribute to the formation of early autophagosomal structures (Guo et al., 2012). In neurons and transfected cell lines, BIN1 has been shown to co-localize with AP-1 at the TGN in a clathrin-dependent manner (Huser et al., 2013). Having also been associated with TGN trafficking in other contexts (Sarret et al., 2004), these observations link BIN1 to autophagosome biogenesis through its involvement in TGN-derived membrane trafficking.

### Transcriptional regulation of autophagy

Microglial BIN1 remodels the autophagy transcriptome in two directions, elevating lysosomal genes *Lamp1*, *Lamp2*, and *Cln3* while simultaneously suppressing core initiators such as *Ulk1*, *Sqstm1*, *Ambra1*, *Map1lc3a*, and several *Atg* genes (Sudwarts et al., 2022). These bidirectional shifts indicate that BIN1 can fine-tune turnover capacity rather than simply turning autophagy “on” or “off.”

A second layer of control is exerted through BIN1’s repression of the proto-oncogene MYC, a transcription factor with context-dependent effects on autophagy. Nuclear BIN1 isoforms (e.g., isoform 9) bind and inhibit MYC, whereas neuronal isoforms do not, pointing to isoform-specific regulation. MYC supports autophagosome formation, c-Myc knock-down lowers LC3-II levels and blocks autophagy initiation (Toh et al., 2013), and can directly activate ULK3, suggesting a potential BIN1–MYC–ULK3 axis (Liu et al., 2020). In Drosophila, both basal and starvation-induced autophagy require c-Myc, highlighting its critical role in cellular adaptation to stress by promoting autophagy (Nagy et al., 2013). However, elevated MYC levels, particularly in cancer, have been associated with the repression of autophagy-related genes and impaired autophagic activity (Cianfanelli et al., 2015; Medda et al., 2023).

Mechanistically, MYC competes with MiT/TFE family members (TFEB, TFE3, MITF, and TFEC) for E-box/CLEAR motifs, dampening lysosomal and autophagy gene expression (Annunziata et al., 2019). MiT/TFE factors are themselves bHLH-Zip proteins that recognize the same motif (Tan et al., 2022), and MYC binds adjacent E-boxes at many of these promoters (Bretones et al., 2015). Histone deacetylase inhibition can relieve this competition, decreasing MYC occupancy and permitting TFEB-driven transcription (Annunziata et al., 2019). This dynamic is seen in cancer and stem cells, where MYC overexpression suppresses lysosomal and autophagic activity, contributing to malignant transformation.

BIN1 adds a stress-responsive switch to this circuitry. Under oxidative conditions, BIN1 sequesters MYC in the cytoplasm (Damian-Zamacona et al., 2018), indirectly favoring TFEB nuclear import; reciprocal MYC–TFEB regulation has been documented in hematologic models (Fernandez et al., 2022). Single-nucleus RNA-seq data place BIN1 downstream of TFEB in Alzheimer’s-linked astrocyte states associated with heightened autophagy and stress responses (Grubman et al., 2019). Together, these findings position BIN1, MYC, and TFEB as an integrated transcriptional node that couples cellular stress cues to autophagy capacity and may influence AD progression.

### Chaperone-mediated autophagy

As described above, BIN1 regulates clathrin-mediated endocytosis and early-to-recycling endosome traffic—processes required for the membrane turnover of LAMP2A, the lysosomal receptor that gates chaperone-mediated autophagy (Braulke and Bonifacino, 2009). Through dynamin-dependent budding and curvature sensing, BIN1 controls cargo return from endosomes to the plasma membrane and reshapes liposomes or plasma-membrane invaginations *in vitro* (Picas et al., 2014; Wu and Baumgart, 2014; Adam et al., 2015). These membrane-remodeling activities likely support the continued delivery of LAMP2A-containing vesicles to lysosomes.”

Consistent with this model, BIN1 depletion in microglia down-regulates core CMA chaperones *Hsc70*, *Hsp90aa1*, *Hsp90ab1*, *Dnaja1*, and *Dnajb1* (Crotti et al., 2019), suggesting that loss of BIN1 compromises both receptor delivery and chaperone availability. Such dual impairment would hinder selective degradation of oxidized or misfolded proteins, potentially accelerating amyloid-β accumulation and neuroinflammation in AD.

### Synaptic autophagy orchestrated by bridging integrator 1 and its partners

At presynaptic terminals, BIN1 collaborates with a cohort of curvature-sensing proteins to couple vesicle cycling to local autophagy. Endophilins, synaptojanin, and the scaffold Bassoon, all BIN1 interactors, drive LC3-positive membrane formation at boutons (Micheva et al., 1997a; Modregger et al., 2003; Okerlund et al., 2017; Vanhauwaert et al., 2017; De Rossi et al., 2020; Yang et al., 2022). Endophilin A isoforms link activity-evoked Ca²⁺ influx to autophagosome biogenesis, while Endophilin B1 engages BECN1/UVRAG; its loss impairs autophagic flux and worsens amyloid-β pathology (Takahashi et al., 2009; Xiao et al., 2015a; Murdoch et al., 2016; Soukup et al., 2016; Hernandez-Diaz et al., 2022; Bademosi et al., 2023). Crucially, BIN1 senses neuronal Ca²⁺ via Ca_v_1.2 and modulates activity-dependent autophagy, providing a direct link between firing rate and proteostatic clearance (Myers et al., 2016; Saha et al., 2024). Together, these interactions place BIN1 at the nexus of synaptic vesicle turnover, membrane remodeling, and autophagy, functions whose disruption may compromise synaptic integrity in AD.

### Cross-disease perspectives: bridging integrator 1 myopathies illuminate autophagy in Alzheimer’s disease

Centronuclear myopathies (CNMs), characterized by centralized muscle fiber nuclei, exhibit autophagic dysfunction linked to mutations in *MTM1*, *DNM2*, and *BIN1* (Merlini et al., 2014). In *BIN1*-dominant CNM, SQSTM1/p62 accumulation in muscle tissue indicates impaired autophagic flux (Bohm et al., 2014). BIN1 interacts with MTM1 and DNM2, forming a triad critical for membrane dynamics and autophagy (Giraud and Laporte, 2024). In *DNM2*-R465W CNM mice, BIN1 overexpression rescues muscle atrophy and T-tubule defects, suggesting BIN1 mitigates DNM2-related autophagic deficits (Durieux et al., 2012; Lionello et al., 2022). Similarly, Mtm1-null mice show increased autophagosomes and reduced autophagic flux, with elevated LC3-I/II and p62 levels (Al-Qusairi et al., 2013; Fetalvero et al., 2013). BIN1 overexpression or MTM1 restoration rescues CNM phenotypes, while DNM2 depletion ameliorates BIN1- or MTM1-related defects, indicating a shared pathway where MTM1 enhances BIN1 function and both regulate DNM2 (Cowling et al., 2014, 2017; Tasfaout et al., 2018; Lionello et al., 2019; Silva-Rojas et al., 2022; Giraud et al., 2023).

DNM2 supports autophagosome formation via interactions with Bif-1 and LC3, mediating vesicle generation and phagophore maturation DNM2 (Takahashi et al., 2016; Riera et al., 2019). The *DNM2*-R465W mutant impairs autophagy by mislocalizing to the plasma membrane (Puri et al., 2020). BIN1 likely synergizes with DNM2 in these processes, and disruptions may contribute to CNM and AD pathologies. Collectively, muscle-disease data highlight a BIN1–MTM1–DNM2 network that balances membrane scission with autophagic flux; understanding this circuitry offers mechanistic clues for how BIN1 safeguards neuronal homeostasis—and how its failure may accelerate AD.

## Conclusion

**Figure 3:**
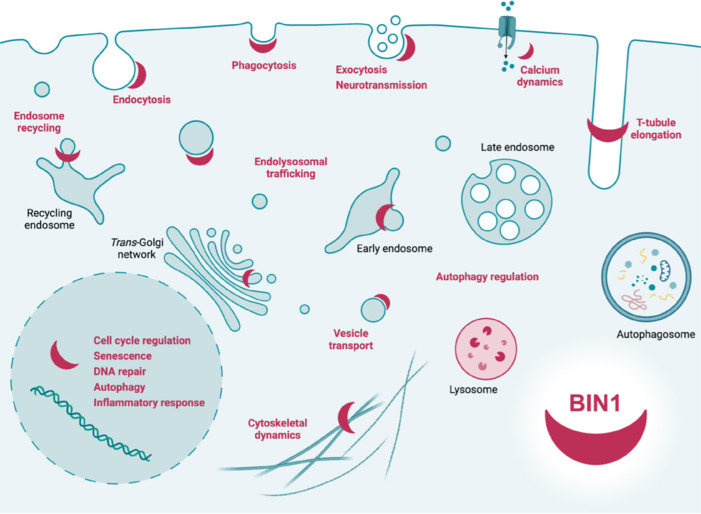
Schematic overview of general bridging integrator 1 (BIN1) functions. BIN1 is primarily associated with endocytosis, endosomal trafficking, and recycling. BIN1 also regulates membrane, cytoskeletal, and microtubule dynamics, as well as autophagy, calcium signaling, synaptic transmission, and inflammation. Nuclear isoforms of BIN1 contribute to DNA repair and regulation of cell cycle progression, autophagy, immune response, and senescence. Created with BioRender.com.

Taken together, the evidence presented underscores the complexity and importance of BIN1 in orchestrating multiple facets of the autophagy and endolysosomal pathways. Initially recognized primarily for its role in membrane remodeling, the influence of BIN1 has now been expanded to encompass a broader regulatory scope, from modulating the transcriptional profiles of autophagy- and endolysosome-related genes to shaping the dynamic protein interactions that govern vesicle formation, maturation, and turnover (**Figure 3**). At the transcriptional level, the ability of BIN1 to fine-tune gene expression may serve as a master switch, determining the balance and abundance of critical autophagic components. Meanwhile, its direct, physical interactions with key proteins in the autophagy and endolysosomal machinery exemplify the capacity of BIN1 to coordinate intricate molecular events on a more immediate, structural scale.

These multifaceted roles place BIN1 in an influential position within cellular homeostasis. Disruptions in its function can reverberate through the cell, leading to suboptimal degradation of protein aggregates, impaired clearance of damaged organelles, and eventual compromise of neuronal integrity — traits that are particularly relevant to neurodegenerative disorders such as AD. By bridging the gap between gene-level regulatory mechanisms and the intricate protein interactions at the heart of vesicular trafficking, BIN1 emerges as a central integrator of autophagic efficiency and cellular resilience.

Looking ahead, a deeper understanding of the tiered influence of BIN1 over autophagy, and how it weaves together transcriptional control with precise molecular assemblies, can open new avenues for targeted therapeutic interventions. Pharmacological or genetic strategies aimed at normalizing BIN1 function could restore autophagic flux and reduce cellular stress. Ultimately, these insights highlight BIN1 as a critical node in the cellular network that safeguards proteostasis and neuronal health, setting the stage for innovative research and potential clinical advances in the management of diseases driven by impaired autophagy.

## Data Availability

*Not applicable*.
